# Long-term reduction in morning and nighttime blood pressure after renal denervation: 36-month results from SPYRAL HTN-ON MED trial

**DOI:** 10.1038/s41440-022-01042-8

**Published:** 2022-10-15

**Authors:** Kazuomi Kario, Felix Mahfoud, David E. Kandzari, Raymond R. Townsend, Michael A. Weber, Roland E. Schmieder, Konstantinos Tsioufis, Stuart Pocock, Sandeep Brar, Douglas A. Hettrick, Martin Fahy, Michael Böhm

**Affiliations:** 1 Jichi Medical University School of Medicine, Tochigi, Japan; 2 Universitätsklinikum des Saarlandes, Saarland University, Homburg/Saar, Germany; 3 Piedmont Heart Institute, Atlanta, GA USA; 4 Perelman School of Medicine, University of Pennsylvania, Philadelphia, PA USA; 5 SUNY Downstate College of Medicine, Brooklyn, NY USA; 6 University Hospital Erlangen, Erlangen, Germany; 7 1st Department of Cardiology, National and Kapodistrian University of Athens, Hippocratio Hospital, Athens, Greece; 8 London School of Hygiene & Tropical Medicine, London, UK; 9 Medtronic, Santa Rosa, CA USA

**Keywords:** Renal denervation, Randomized controlled trial, Resistant hypertension, Cardiovascular prognosis

## Abstract

Elevated morning and nighttime blood pressures (BP) are associated with increased risk of cardiovascular events such as stroke and myocardial infarction. We compared the long-term changes in morning and nighttime BP in patients with uncontrolled hypertension (office systolic BP between 150 and <180 mmHg/diastolic BP ≥ 90 mmHg; mean ambulatory systolic BP (SBP) between 140 and <170 mmHg; 1–3 prescribed antihypertensive medications). Eighty patients were randomized to RDN or sham control. In patients taking at least 3 antihypertensive medications at 36 months (*N* = 23 RDN group; *N* = 23 sham group), the 24 h ambulatory SBP as well as morning (7:00–9:00AM) and nighttime (1:00–6:00AM) ambulatory SBP were significantly lower for the RDN group compared to sham control (24 h SBP: −20.2 vs. −10.2, *p* = 0.0087; morning SBP: −23.9 vs. −8.0 mmHg, *p* = 0.029; nighttime SBP: −20.8 vs. −7.2 mmHg, *p* = 0.0011). At 36 months, 24 h SBP was controlled to <130 mmHg in 40% of RDN patients in the morning compared to 6% for the sham group; *P* = 0.021 and in 80% of the RDN patients at night compared to 39% in the sham group; *P* = 0.019. Major adverse events through 36 months were rare in both groups, and there were no renal artery re-interventions or vascular complications. Morning and nighttime SBP were significantly lower in patients prescribed at least 3 antihypertensive medications at 36 months in the SPYRAL HTN-ON MED trial for RDN compared with sham control. The results suggest RDN has significant benefit when the risk of cardiovascular events is highest.

## Abstract (web summary)

Always-on” 24 h blood pressure (BP) lowering effect of renal denervation with sufficient reduction of nighttime and morning BP that is difficult to control even by office BP-guided intense titration of multiple antihypertensive drugs.

## Graphical abstract

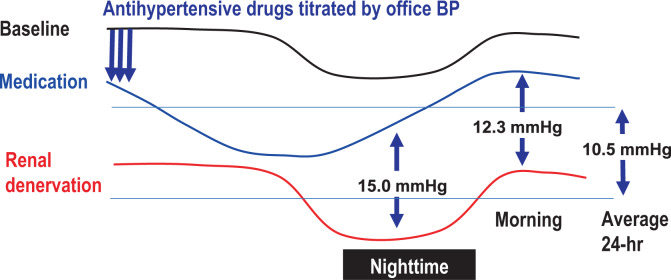

## Introduction

Autonomically modulated circadian variability in arterial blood pressures (BP) is associated with both overall cardiovascular risk and the timing of adverse clinical events [1–5]. Indeed, elevated morning [6, 7] and nighttime [8–14] BP are each independently associated with increased risk of cardiovascular events including myocardial infarction and stroke. Hence, morning and nighttime hypertension management is critical to achieve “ideal 24 h BP control” [15]. Unfortunately, antihypertensive medication blood levels may reach a relative nadir during the pre-waking period due to once daily (often morning) dosing schedules and the pharmacokinetics of drug clearance [16]. Furthermore, those with apparent treatment resistant hypertension, defined as uncontrolled blood pressure despite prescription of ≥3 antihypertensive medication, are at increased risk [17]. Recent studies have demonstrated that true resistant hypertension including both uncontrolled office BP and out-of-office BPs detected by ambulatory BP monitoring or home BP monitoring, was associated with increased cardiovascular risk [18–20]. Nighttime BP was especially associated with cardiovascular events compared to daytime BP in resistant hypertension patients [18, 19].

Randomized sham-controlled trials have demonstrated the safety and efficacy of radiofrequency catheter-based renal denervation (RDN) to lower blood pressure in both the presence [21, 22] and absence [23, 24] of concomitant drug therapy. We recently reported favorable long-term safety and efficacy results at 3 years in the SPYRAL HTN ON-MED trial [25]. During long-term follow-up, up-titration of medication guided by office BP may reduce office and daytime BP but may still be insufficient to reduce nighttime and morning BP prior to taking morning pills. This report investigates morning and nighttime BP changes in patients prescribed ≥3 antihypertensive drugs at 36 months post randomization in order to determine whether (1) RDN persistently decreases 24 h BP (including nighttime and morning) and (2) whether the differences between RDN and control groups (treated by drug treatment only) will be greater in resistant hypertensive patients during long-term follow-up including up-titration of antihypertensive medication guided by office BP.

## Methods

The SPYRAL HTN-ON MED trial is an international, prospective, randomized, blinded, sham-controlled trial that has been previously described [22, 25]. Briefly, patients were enrolled with office systolic BP (SBP) ≥ 150 mmHg and <180 mmHg, office diastolic BP (DBP) ≥90 mmHg, mean 24 h SBP ≥140 mmHg and <170 mmHg, and prescribed 1–3 antihypertensive medications at baseline. Patients were randomized 1:1 to RDN or sham control. The primary endpoint was the treatment difference in mean 24 h SBP at 6 months between RDN and sham control groups. Patients and designated trial staff conducting follow-up assessments were blinded to randomization through 12 months. All patients provided written informed consent to participate, and the trial protocol was approved by the local ethics committee or institutional review board. The trial was designed in accordance with the Declaration of Helsinki.

### Study procedures

The four-electrode radiofrequency Symplicity Spyral catheter and the Symplicity G3 radio frequency generator (Medtronic; Minneapolis, MN, USA) were used to produce ablations in a spiral pattern in the main renal arteries, accessory, and branch vessels between 3 and 8 mm in diameter. Eligible patients randomized to the sham procedure remained on the procedure table for at least 20 min following the renal angiogram to ensure blinding. Before initiating 24 h ambulatory blood pressure monitoring at 3, 6, 12, 24, and 36 months (Mobil-O-Graph; IEM; Stolberg, Germany), all subjects ingested prescribed antihypertensive medications in the presence of staff. Antihypertensive medications changes were allowed after the primary 6 months follow up by physician discretion. The study was originally designed to only collect urine and blood tests at baseline and 6 months and, in some patients, at 24 and 36 months following a study protocol update. Duplex ultrasound, CT, or MRI was recommended to be performed either at 12, 24, or 36 months to assess renal artery anatomy. Patients in the sham control group were eligible for crossover to receive the RDN procedure after their 12-month follow-up visit according to patient and investigator discretion.

### Study endpoints

Long-term safety was compared between RDN and sham control groups through 36 months using a composite endpoint of major adverse events. Long-term efficacy was evaluated from baseline to 36 months. Nighttime (1:00–6:00 AM) and morning (7:00–9:00 AM) BPs were separately calculated from the average of BPs. Morning BP parameters were defined as follows: average morning SBP (average of morning SBPs), moving peak morning SBP (highest 1 h moving average of 3 consecutive SBPs over the morning period) and minimum (lowest recorded value) and maximum (highest recorded value during the morning interval) morning SBP. Nighttime BP parameters were similarly defined as follows: average nighttime SBP (average of nighttime interval SBPs), average peak night-time SBP (average of the 3 highest measurements over the nighttime interval) and minimum (lowest recorded value) and maximum (highest recorded value during the nighttime interval) nighttime SBP.

### Statistical analysis

Statistical analyses were performed on the subgroup of patients prescribed ≥3 antihypertensive drugs at the time of the 36-month follow up. Continuous variables are reported as mean (SD). Categorical variables are reported as counts and percentages and were compared between treatment groups using exact binomial tests. Baseline measures were compared between the groups using *t*-tests. Follow-up change measures were compared between the RDN group and sham control group using ANCOVA, adjusting for baseline measurements. The last observations of blood pressure measurements and laboratory values were used to impute 36-month values for patients who crossed over from the sham group and received RDN.

## Results

**Fig. 1:**
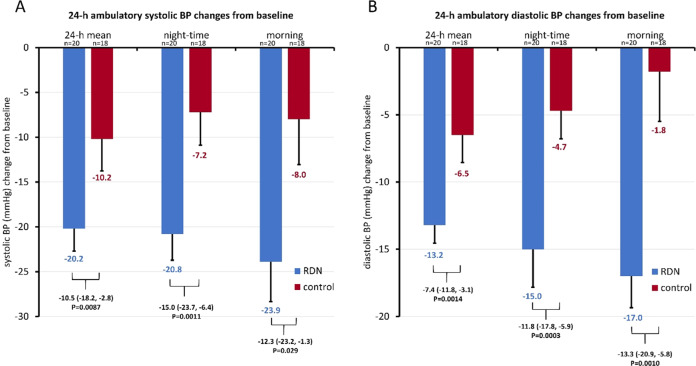
Reduction in 24 h mean, night-time, and morning ambulatory (**A**) systolic and (**B**) diastolic BP at 36 months from baseline comparing patients prescribed ≥3 more medications from the RDN and sham control group. Blood pressure treatment differences from baseline comparisons between RDN and control patients are ANCOVA adjusted for baseline BP. Morning is defined as 01:00–06:00 h, and nighttime is defined as 07:00–09:00 h. 36-month sham control blood pressures include 7 imputed values from crossover patients

Between July 22, 2015, and June 14, 2017, 80 patients were randomly assigned to undergo RDN (*n* = 38) or a sham control (*n* = 42). Baseline demographic data were similar for the subgroup of RDN (*n* = 23), and sham group (*n* = 23) patients prescribed at least 3 antihypertensive classes of antihypertensive medication at 36 months and were also comparable with the full study cohort (Table 1). The average number of prescribed medications in the ≥3 medication group was 3.7 ± 0.7 for the RDN group and 3.9 ± 1.2 in the control group (*p* = 0.49; Table 2). Baseline morning (7:00–9:00 AM) and nighttime (1:00–6:00 AM) ambulatory SBP measurements for the subgroup were similar between RDN and sham control. Similar to the overall results, reductions in 24 h SBP in patients on at least 3 antihypertensive meds was significantly greater for RDN when compared to sham (−20.2 vs. −10.2, *p* = 0.0087). Both morning and nighttime SBP were significantly reduced from baseline in the RDN group compared with sham control (morning SBP: −23.9 vs. −8.0 mmHg, *p* = 0.029; nighttime SBP: −20.8 vs. −7.2 mmHg, *p* = 0.0011; Fig. 1). At 36 months, 24 h SBP was controlled to <130 mmHg in 40% (8/20) of RDN patients in the morning compared to 6% (1/18) for the sham group; *p* = 0.021 and in 80% of the RDN (16/20) group patients at night as compared to 39% (7/18) in the sham group; *p* = 0.019. Office SBP decreased from baseline by −21.3 ± 14.7 mmHg in the RDN group and by −12.2 ± 26.7 mmHg in the control group (between group difference −5.9 (95% CI:−19.0,7.0; *p* = 0.367).

**Table 1:** Baseline characteristics for SPYRAL HTN ON-MED patients prescribed ≥ 3 antihypertensive drugs at 36 months

Patient demographics	RDN	CONTROL	*P* VALUE
Age (years)	55.1 ± 8.8 (23)	51.0 ± 10.2 (23)	0.1456
Female	4.3% (1/23)	17.4% (4/23)	0.3463
Male	95.7% (22/23)	82.6% (19/23)	0.3463
Race			
White	39.1% (9/23)	34.8% (8/23)	1.0000
Black or African American	8.7% (2/23)	17.4% (4/23)	0.6652
BMI (kg/m^2^)	32.3 ± 5.4 (23)	33.8 ± 4.5 (23)	0.3258
Estimated glomerular filtration rate (ml/min/1·73 m²)	80.1 ± 15.1 (23)	81.0 ± 19.1 (23)	0.8605
Type 2 Diabetes	8.7% (2/23)	30.4% (7/23)	0.1346
Current Smoker	21.7% (5/23)	26.1% (6/23)	1.0000
History of Sleep Apnea	17.4% (4/23)	39.1% (9/23)	0.1894
History of Coronary Artery Disease	0.0% (0/23)	4.3% (1/23)	1.0000
History of Peripheral Artery Disease	0.0% (0/23)	0.0% (0/23)	NA
History of Stroke	0.0% (0/23)	0.0% (0/23)	NA
*Blood Pressure*			
Office systolic blood pressure	166.0 ± 5.9 (23)	162.1 ± 7.5 (23)	0.0595
Office diastolic blood pressure	99.6 ± 7.1 (23)	102.0 ± 7.4 (23)	0.2831
Mean 24 h systolic blood pressure	151.8 ± 6.1 (23)	152.4 ± 6.9 (22)	0.7512
Mean 24 h diastolic blood pressure	96.7 ± 7.4 (23)	98.9 ± 7.1 (22)	0.3296
Morning systolic blood pressure	156.6 ± 13.7 (23)	155.0 ± 16.9 (22)	0.7342
Morning diastolic blood pressure	102.4 ± 10.9 (23)	102.0 ± 14.7 (22)	0.9082
Nighttime systolic blood pressure	141.7 ± 13.8 (23)	142.8 ± 9.2 (22)	0.7457
Nighttime diastolic blood pressure	89.1 ± 14.1 (23)	91.4 ± 9.4 (22)	0.5368

*P* values from exact binomial tests for categorical data or *t*-tests for continuous data

**Table 2:** Prescribed antihypertensive drug classes and medication burden for SPYRAL HTN ON-MED patients prescribed ≥ 3 antihypertensive drugs at 36 months

	RDN ± SD (*n* = 23)	control ± SD (*n* = 23)	ANCOVA difference (95% CI)	*p* value
Number of AH medications at 36 months	3.7 ± 0.7	3.9 ± 1.2	−0.2 (−0.8,0.4)	0.49
AH medication burden at 36 months	10.5 ± 5.6	15.9 ± 18.6	−5.2 (−13.3,2.8)	0.20

Antihypertensive medication burden is a composite index based on the doses of medications but multiplies this result by the number of prescribed medications. Antihypertension medication burden is calculated for all antihypertensive medication prescribed at study specified follow-up visits for each patient and added to yield a single, summative score. All classes of drug are considered equivalent in potency so the “class weight” is set a “1” for all antihypertensive medications

$$MedIndex2 = no.of\,meds\mathop {\sum}\nolimits_{AH\,Meds} {\left( {class\,weight\frac{{prescribed\,dose}}{{standard\,dose}}} \right)}$$

Class weight = 1

Standard dose = JNC 7 max daily recommended dose

*SD* standard deviation, *CI* confidence interval, *AH* antihypertensive

**Fig. 2:**
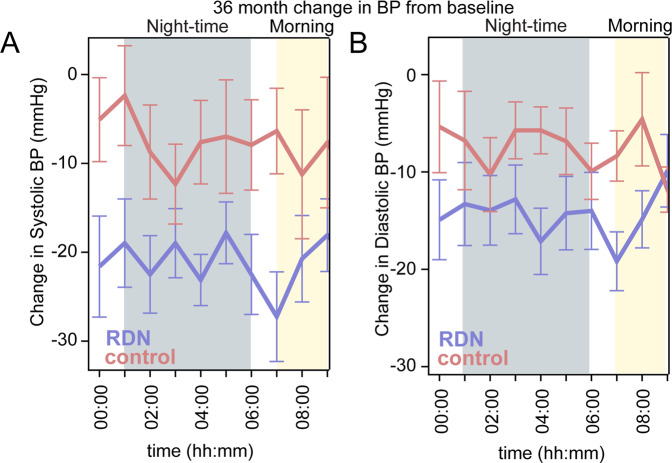
Hourly plots comparing night-time and morning (**A**) systolic and (**B**) diastolic BP at baseline and 36 months post-procedure between patients prescribed ≥3 medications from the RDN and sham control group. RDN: blue; control: salmon. Error bars represent standard error. Nighttime is defined as between 01:00 h and 06:00 h, morning is defined as between 07:00 h and 09:00 h. 36-month sham control blood pressures include 7 imputed values from crossover patients

**Fig. 3:**
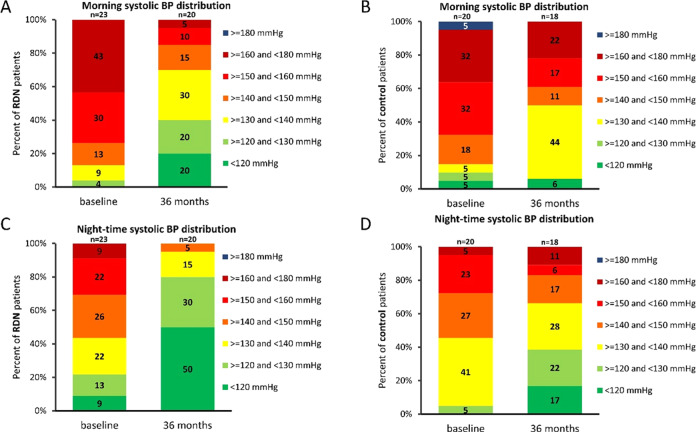
Distribution of morning and nighttime 24 h ambulatory systolic BP at baseline and 36 months in **A** and **C** RDN patients prescribed ≥3 medications, and **B** and **D** control patients prescribed ≥3 medications. 36-month sham control blood pressures include 7 imputed values from crossover patients

The circadian systolic and diastolic 24 h blood pressure curves decreased significantly in both groups from randomization to 36 months (Fig. 2). However, the decrease in circadian BP was greater throughout the day and night in the RDN group compared to control. Similarly, the proportion of patients achieving various lower blood pressure levels during the morning and nighttime periods increased in both groups at 36 months, but the proportions were more favorable in the RDN group (Fig. 3 and supplementary Fig. 1).

**Fig. 4:**
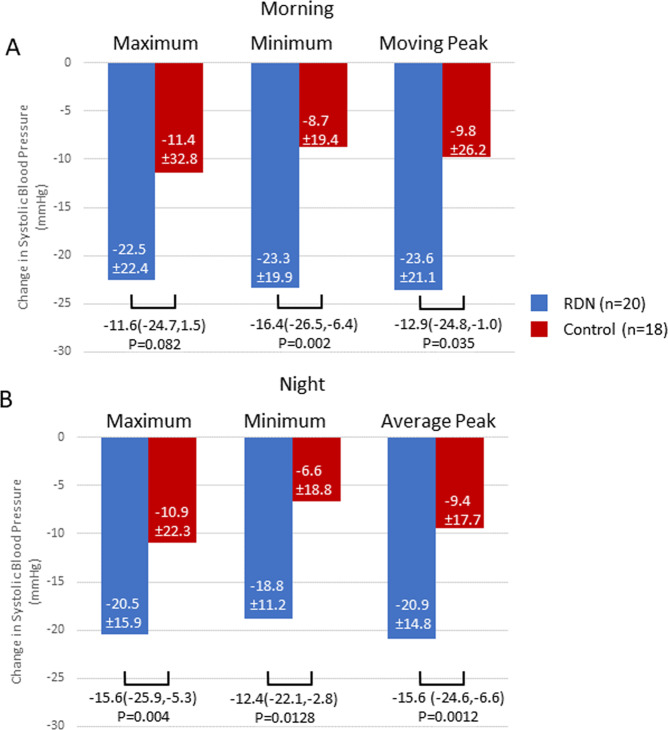
Changes in maximum, minimum and moving peak systolic blood pressure between baseline and 36 months follow up during the morning (**A**) and Nighttime (**B**) for RDN (blue) and control (red) patients prescribed ≥3 medications

Reductions in morning and nighttime minimum and moving peak blood pressure from baseline to 36 months were significantly greater in the RDN group compared to control (Fig. 4). The 36-month maximum nighttime SBP change was also significantly greater in the RDN group while morning maximum changes also trended lower for in the RDN group (Fig. 4).

As previously reported, major adverse events through 36 months were rare in both groups, and no renal artery re-interventions or vascular complications were observed [26].

## Discussion

This is the first report to show significant and persistent nighttime and morning BP reduction following radiofrequency RDN compared to a sham control group at 3 years. These outcomes were consistent irrespective of the number of medications that were prescribed, including high risk patients on 3 or more antihypertensive medications. Whereas control group patients had somewhat variable long-term BP reductions over 24 h, presumably due to increased drug usage guided by office BP, RDN group patients had more consistent BP reductions across the night and morning periods. This indicates that long term up-titration of medication reduced office and daytime BPs, but it’s BP lowering effect appears insufficient to achieve nighttime and morning BP control. However, RDN significantly and persistently reduced 24 h BPs including nighttime and morning BP, providing more uniform BP control over a 24 h period.

Ambulatory blood pressure, particularly nighttime and morning surge blood pressure predict cardiovascular outcomes including heart failure, stroke, and mortality better than clinic blood pressure [2, 3, 8, 10, 14]. Recently, the JAMP trial (Japan Ambulatory Blood Pressure Monitoring Prospective) showed that higher nighttime blood pressure was strongly associated with increased cardiovascular risk, and especially heart failure, within a population of over 6500 elderly patients with 24 h BP monitoring [4]. Also, a recent analysis of the real world Global SYMPLICITY registry, including over 3000 patients showed that radiofrequency RDN-induced reductions in blood pressure “time in target range” were associated with fewer strokes and other adverse cardiovascular events within 3 years [27]. We also previously reported consistently lower BP at night and throughout the morning surge period 3 months following RDN in patients off antihypertensive drug therapy in the SPYRAL HTN OFF-MED trial [28].

The definition for morning blood pressure applied in the present analysis was restricted (6:00–8:59 AM) since early morning SBP is the strongest predictor of cardiovascular events [12, 29]. Likewise, nighttime BP was also restricted (1:00–5:59 AM), based on recent European Society of Hypertension practice guidelines for ambulatory blood pressure monitoring [30]. These restrictions are intended to reduce the variability introduced by individual patient sleep-time behavior patterns and thus may better define sleep ambulatory BP. Twenty-four-hour clock-defined nighttime BP has been found to be slightly higher than sleep ambulatory BP [31] and might provide inaccurate representation of sleeping BP when the time interval is too long. The present analysis demonstrated a distinct pattern of circadian BP reduction associated with radiofrequency RDN (Fig. 3) including significant improvement in morning and nighttime minimum and mean moving peak SBP as well as maximum nighttime SBP, compared with control patients (Fig. 4). Taken together, these observations highlight the importance of antihypertensive strategies targeting nighttime SBP and might broaden the benefit of RDN across the spectrum of hypertensive populations.

The magnitude of BP reduction following RDN may be more apparent at nighttime or in the early morning hours. Notably, the between-group SBP changes were numerically lower for daytime but not nighttime SBP in the SPYRAL HTN OFF MED pivotal trial [28]. In the present analysis, the magnitude of the difference between RDN and control was nominally greater for nighttime and morning BP compared to daytime and 24 h SBP (Fig. 1), primarily due to greater variability in BP in the sham vs. RDN group. Note also in Fig. 1 the smaller range of mean SBP reduction in the RDN group between morning and nighttime (−19.6 to −23.9 mmHg) as compared to the sham group (−7.2 to −13.7 mmHg). This wider range observed in the sham group seems consistent with common once-daily morning dosing of antihypertensive drugs and these might be less effective during sleeping hours when plasma levels of drug concentrations are the lowest. Thus, radiofrequency RDN addresses critical challenges associated with antihypertensive drugs pharmacokinetics, even when patients are adherent to prescribed dosing regimens suggesting that RDN is “always-on” at times when it’s needed most.

The present results supporting RDN efficacy to at least 3 years of follow-up confirm and extend recent pre-clinical [32, 33] and clinical trial [25, 34–37] reports of long-term safety and efficacy of radiofrequency RDN. Note that no repeat procedures have been performed to date in the SPYRAL HTN trials.

The present analysis has limitations. Non-adherence to prescribed antihypertensive drug therapy was objectively assessed at discrete timepoints but adherence over an extended period is uncertain. BP control worsened in the US during the COVID-19 pandemic which may have impacted BP results [38]. However, in-person follow-up visits were performed despite pandemic restrictions. The applied definitions of morning and nighttime periods were restricted to ensure a greater likelihood that nighttime was specific to sleeping hours and were based on prior publications in which these specific time periods were more predictive of impact of treatment on cardiovascular risk [31]. Also, the SPYRAL HTN trials had more rigorous requirements for acquisition of 24 h BP data and only records with at least 21 daytime and 12 valid nighttime measurements were accepted.

In summary, morning and nighttime SBP were significantly reduced in patients prescribed at least 3 antihypertensive medications at 36 months in the SPYRAL HTN-ON MED trial after RDN compared to sham control. The results during these times of high sympathetic tone suggest that radiofrequency RDN has significant long-term benefit when the plasma levels of drug concentrations are the lowest and the risk of cardiovascular events is highest.

## Supplementary information

Supplementary Materials

## References

[1] Kario K, Saito I, Kushiro T, Teramukai S, Tomono Y, Okuda Y (2016). Morning Home Blood Pressure Is a Strong Predictor of Coronary Artery Disease: The HONEST Study. J Am Coll Cardiol.

[2] Kario K, Pickering TG, Umeda Y, Hoshide S, Hoshide Y, Morinari M (2003). Morning surge in blood pressure as a predictor of silent and clinical cerebrovascular disease in elderly hypertensives: a prospective study. Circulation.

[3] Li Y, Thijs L, Hansen TW, Kikuya M, Boggia J, Richart T (2010). Prognostic value of the morning blood pressure surge in 5645 subjects from 8 populations. Hypertension.

[4] Kario K, Hoshide S, Mizuno H, Kabutoya T, Nishizawa M, Yoshida T (2020). Nighttime Blood Pressure Phenotype and Cardiovascular Prognosis: Practitioner-Based Nationwide JAMP Study. Circulation.

[5] Kario K, Hoshide S, Mizuno H, Kabutoya T, Nishizawa M, Yoshida T, et al. Nighttime hemodynamic phenotype. A novel risk factor for cardiovascular disease, especially heart failure: the practitioner-based nationwide JAMP study. Clin Res Cardiol. 2022. 10.1007/s00392-022-02051-w. [Epub ahead of print]. 10.1007/s00392-022-02051-w 35760927

[6] Hoshide S, Yano Y, Haimoto H, Yamagiwa K, Uchiba K, Nagasaka S (2016). Morning and Evening Home Blood Pressure and Risks of Incident Stroke and Coronary Artery Disease in the Japanese General Practice Population: The Japan Morning Surge-Home Blood Pressure Study. Hypertension.

[7] Kario K, Ishikawa J, Pickering TG, Hoshide S, Eguchi K, Morinari M (2006). Morning hypertension: the strongest independent risk factor for stroke in elderly hypertensive patients. Hypertens Res.

[8] Dolan E, Stanton A, Thijs L, Hinedi K, Atkins N, McClory S (2005). Superiority of ambulatory over clinic blood pressure measurement in predicting mortality: the Dublin outcome study. Hypertension.

[9] Sega R, Facchetti R, Bombelli M, Cesana G, Corrao G, Grassi G (2005). Prognostic value of ambulatory and home blood pressures compared with office blood pressure in the general population: follow-up results from the Pressioni Arteriose Monitorate e Loro Associazioni (PAMELA) study. Circulation.

[10] Ben-Dov IZ, Kark JD, Ben-Ishay D, Mekler J, Ben-Arie L, Bursztyn M (2007). Predictors of all-cause mortality in clinical ambulatory monitoring: unique aspects of blood pressure during sleep. Hypertension.

[11] Kario K (2018). Nocturnal Hypertension: New Technology and Evidence. Hypertension.

[12] Fujiwara T, Hoshide S, Kanegae H, Kario K (2020). Cardiovascular Event Risks Associated With Masked Nocturnal Hypertension Defined by Home Blood Pressure Monitoring in the J-HOP Nocturnal Blood Pressure Study. Hypertension.

[13] Hoshide S, Kanegae H, Kario K (2021). Nighttime home blood pressure as a mediator of N-terminal pro-brain natriuretic peptide in cardiovascular events. Hypertens Res.

[14] Yang WY, Melgarejo JD, Thijs L, Zhang ZY, Boggia J, Wei FF (2019). Association of Office and Ambulatory Blood Pressure With Mortality and Cardiovascular Outcomes. JAMA.

[15] Kario K (2017). Perfect 24-h management of hypertension: clinical relevance and perspectives. J Hum Hypertens.

[16] Kario K (2005). Time for focus on morning hypertension: pitfall of current antihypertensive medication. Am J Hypertens.

[17] Carey RM, Calhoun DA, Bakris GL, Brook RD, Daugherty SL, Dennison-Himmelfarb CR (2018). Resistant Hypertension: Detection, Evaluation, and Management: A Scientific Statement From the American Heart Association. Hypertension.

[18] Kario K, Hoshide S, Narita K, Okawara Y, Kanegae H (2021). Investigators n. Cardiovascular Prognosis in Drug-Resistant Hypertension Stratified by 24-H Ambulatory Blood Pressure: the JAMP Study. Hypertension.

[19] Narita K, Hoshide S, Kario K (2022). Nighttime Home Blood Pressure Is Associated With the Cardiovascular Disease Events Risk in Treatment-Resistant Hypertension. Hypertension.

[20] Kario K, Mogi M, Hoshide S (2022). Latest hypertension research to inform clinical practice in Asia. Hypertens Res.

[21] Azizi M, Sapoval M, Gosse P, Monge M, Bobrie G, Delsart P (2015). Optimum and stepped care standardised antihypertensive treatment with or without renal denervation for resistant hypertension (DENERHTN): a multicentre, open-label, randomised controlled trial. Lancet.

[22] Kandzari DE, Bohm M, Mahfoud F, Townsend RR, Weber MA, Pocock S (2018). Effect of renal denervation on blood pressure in the presence of antihypertensive drugs: 6-month efficacy and safety results from the SPYRAL HTN-ON MED proof-of-concept randomised trial. Lancet.

[23] Townsend RR, Mahfoud F, Kandzari DE, Kario K, Pocock S, Weber MA (2017). Catheter-based renal denervation in patients with uncontrolled hypertension in the absence of antihypertensive medications (SPYRAL HTN-OFF MED): a randomised, sham-controlled, proof-of-concept trial. Lancet.

[24] Böhm M, Kario K, Kandzari DE, Mahfoud F, Weber MA, Schmieder RE (2020). Efficacy of catheter-based renal denervation in the absence of antihypertensive medications (SPYRAL HTN-OFF MED Pivotal): a multicentre, randomised, sham-controlled trial. Lancet.

[25] Mahfoud F, Kandzari DE, Kario K, Townsend RR, Weber MA, Schmieder RE (2022). Long-term efficacy and safety of renal denervation in the presence of antihypertensive drugs (SPYRAL HTN-ON MED): a randomised, sham-controlled trial. Lancet.

[26] Heradien M, Mahfoud F, Greyling C, Lauder L, van der Bijl P, Hettrick DA, et al. Renal denervation prevents subclinical atrial fibrillation in patients with hypertensive heart disease: Randomized, sham-controlled trial. Heart Rhythm. 2022. 10.1016/j.hrthm.2022.06.031. [Epub ahead of print]. 10.1016/j.hrthm.2022.06.031 35781044

[27] Felix Mahfoud GM, Roland S, Luis R, Markus S, Krzystof N, Bryan W, et al. Blood pressure and MACE reductions after renal denervation: 3-year GSR results. EuroPCR; Paris, France. https://www.pcronline.com/Cases-resources-images/Resources/Course-videos-slides/2022/EuroPCR-2022-Hotlines-Late-Breaking-Trials-in-hypertension-management-SPYRAL-HTN-ON-MED-radiance-HTN-SOLO-and-TRIO-and-more2022.

[28] Kario K, Bohm M, Mahfoud F, Townsend RR, Weber MA, Patel M (2018). Twenty-Four-Hour Ambulatory Blood Pressure Reduction Patterns After Renal Denervation in the SPYRAL HTN-OFF MED Trial. Circulation.

[29] O’Brien E, Parati G, Stergiou G, Asmar R, Beilin L, Bilo G (2013). European Society of Hypertension position paper on ambulatory blood pressure monitoring. J Hypertens.

[30] Parati G, Stergiou G, O’Brien E, Asmar R, Beilin L, Bilo G (2014). European Society of Hypertension practice guidelines for ambulatory blood pressure monitoring. J Hypertens.

[31] Ishikawa J, Hoshide S, Eguchi K, Ishikawa S, Shimada K, Kario K (2012). Nighttime home blood pressure and the risk of hypertensive target organ damage. Hypertension.

[32] Rousselle SD, Brants IK, Sakaoka A, Hubbard B, Jackson ND, Wicks JR (2015). Neuromatous regeneration as a nerve response after catheter-based renal denervation therapy in a large animal model: immunohistochemical study. Circ Cardiovasc Interv.

[33] Sharp ASP, Tunev S, Schlaich M, Lee DP, Finn AV, Trudel J (2022). Histological evidence supporting the durability of successful radiofrequency renal denervation in a normotensive porcine model. J Hypertens.

[34] Volz S, Spaak J, Elf J, Jagren C, Lundin C, Stenborg A (2018). Renal sympathetic denervation in Sweden: a report from the Swedish registry for renal denervation. J Hypertens.

[35] Panchavinnin P, Wanthong S, Roubsanthisuk W, Tresukosol D, Buranakitjaroen P, Chotruangnapa C (2022). Long-term outcome of renal nerve denervation (RDN) for resistant hypertension. Hypertens Res.

[36] Zeijen VJM, Feyz L, Nannan Panday R, Veen K, Versmissen J, Kardys I, et al. Long-term follow-up of patients undergoing renal sympathetic denervation. Clin Res Cardiol. 2022. 10.1007/s00392-022-02056-5. [Epub ahead of print]. 10.1007/s00392-022-02056-5 PMC9622524 35851428

[37] Mahfoud F, Bohm M, Schmieder R, Narkiewicz K, Ewen S, Ruilope L (2019). Effects of renal denervation on kidney function and long-term outcomes: 3-year follow-up from the Global SYMPLICITY Registry. Eur Heart J.

[38] Laffin LJ, Kaufman HW, Chen Z, Niles JK, Arellano AR, Bare LA (2022). Rise in Blood Pressure Observed Among US Adults During the COVID-19 Pandemic. Circulation.

