# Supplementary material for: Long-term reduction in morning and nighttime blood pressure after renal denervation: 36-month results from SPYRAL HTN-ON MED trial
Source: Hypertens Res. 2022 Oct 15;46(1):280–8. doi: 10.1038/s41440-022-01042-8 (PMC9747613; doi:10.1038/s41440-022-01042-8)

**Supplementary Materials:**

**Supplementary Figure 1. Distribution of morning and night-time 24-h ambulatory diastolic BP at baseline and 36 months in A) and C) RDN patients on ≥3 medications, and B) and D) control patients on ≥3 medications**


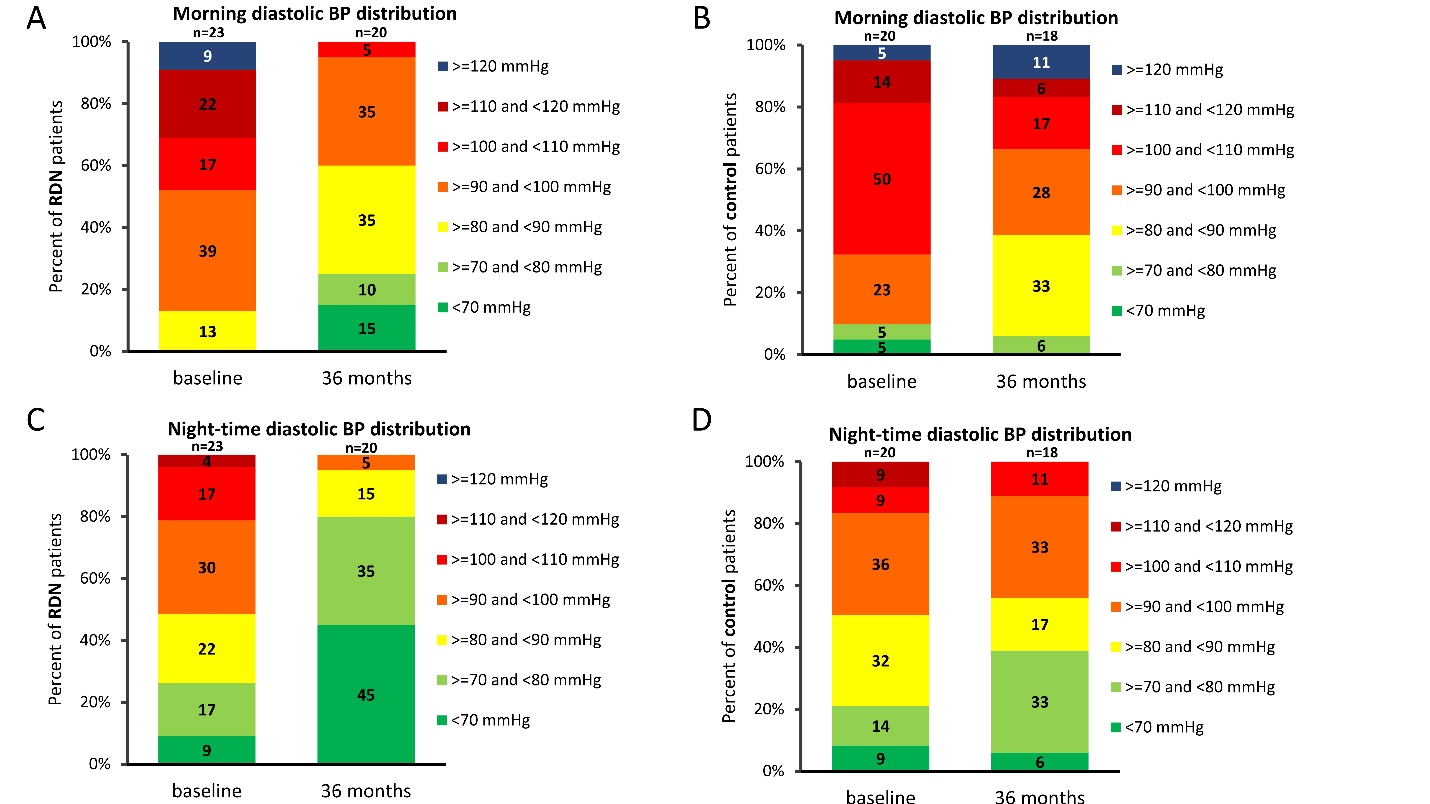

Supplement: Supplementary file 1 — Supplementary Materials [file 41440_2022_1042_MOESM1_ESM.docx]
